# The oxidative stress response of the filamentous yeast *Trichosporon cutaneum* R57 to copper, cadmium and chromium exposure

**DOI:** 10.1080/13102818.2014.965020

**Published:** 2014-10-21

**Authors:** Nevena Lazarova, Ekaterina Krumova, Tsvetanka Stefanova, Nelly Georgieva, Maria Angelova

**Affiliations:** ^a^ Department of Biotechnology, University of Chemical Technology and Metallurgy, 8 Kliment Ohridsky, 1756Sofia, Bulgaria; ^b^ Department of Mycology, The Stephan Angeloff Institute of Microbiology, Bulgarian Academy of Sciences, Academician G. Bonchev 26, 1113Sofia, Bulgaria; ^c^ Department of Immunology, The Stephan Angeloff Institute of Microbiology, Bulgarian Academy of Sciences, Academician G. Bonchev 26, 1113Sofia, Bulgaria

**Keywords:** filamentous yeast, oxidative stress, heavy metals, antioxidant defence, biomarkers of oxidative stress

## Abstract

Despite the intensive research in the past decade on the microbial bioaccumulation of heavy metals, the significance of redox state for oxidative stress induction is not completely clarified. In the present study, we examined the effect of redox-active (copper and chromium) and redox-inactive (cadmium) metals on the changes in levels of oxidative stress biomarkers and antioxidant enzyme defence in *Trichosporon cutaneum* R57 cells. This filamentous yeast strain showed significant tolerance and bioaccumulation capability of heavy metals. Our findings indicated that the treatment by both redox-active and redox-inactive heavy metal induced oxidative stress events. Enhanced concentrations of Cu^2+^, Cr^6+^ and Cd^2+^ caused acceleration in the production of reactive oxygen species (ROS), increase in the level of oxidatively damaged proteins and accumulation of reserve carbohydrates (glycogen and trehalose). Cell response against heavy metal exposure also includes elevation in the activities of antioxidant enzymes, superoxide dismutase and catalase, which are key enzymes for directly scavenging of ROS. Despite the mentioned changes in the stress biomarkers, *T. cutaneum* did not show a significant growth diminution. Probably, activated antioxidant defence contributes to the yeast survival under conditions of heavy metal stress.

## Introduction

Metal ions are considered very important and at the same time very toxic for living organisms. They are some of the main pollutants in the environment. Heavy metals are present in soils as free or exchangeable metal ions, soluble metal complexes, organically bound metals, precipitated or insoluble compounds (oxides, carbonates and hydroxides).[1] Recently, microbial systems, like fungi, bacteria and algae, have been successfully used as adsorbing agents for removal of heavy metals.[2,3] Different species of *Aspergillus*, *Pseudomonas*, *Sporophyticus*, *Bacillus*, *Phanerochaete*, etc. have been reported as efficient reducers.[4] The response of microorganisms towards toxic heavy metals is critically important in the reclamation of polluted sites. Living organisms exposed environmentally to high metal concentrations follow various mechanisms to counter potential toxicity. Among the group of microorganisms used for bioremediation, yeasts are having a leading place.[5]

A possible consequence of heavy metal exposure is an increased production of reactive oxygen species (ROS) such as hydroxyl radical (HO^·^), superoxide radical (**^•^**O_2_^−^) or hydrogen peroxide (H_2_O_2_) that could induce or exacerbate intracellular oxidative stress. These ROS may lead to the unspecific oxidation of proteins and membrane lipids or may cause DNA damage.[6] Defence mechanisms which counteract the impact of ROS, including enzyme and non-enzyme antioxidant systems, are found in all aerobic cells. Interaction between chemical elements, the level of oxidative stress and antioxidant defence play an important role in ecotoxicological response of microorganisms in polluted environments.[7]

The high potential of *Trichosporon cutaneum* strain R57 for heavy metal removal from contaminated waste water has been demonstrated in several previous investigations, showing significant tolerance and bioaccumulation capability for chromium (Cr), cadmium (Cd) and copper (Cu).[8,9] Our previous study has also shown high ability of the same yeast strain to grow in the media supplemented with high content of phenols and resistant to toxic chemicals, such as benzyl alcohol,[10,11] revealed that the capacity of the strain to sustain toxic concentrations of heavy metals in the medium often refers to its ability to accumulate harmful ions in the cells.[12] The question arises regarding the relationship between redox state of the metals and the oxidative stress induction in the yeast cells. Copper and chromium as redox-active metals generate ROS through redox cycling reactions.[13] Redox-inactive Cd impairs antioxidant defences, especially those involving thiol-containing antioxidants and enzymes.

Copper has a dual role in terms of regulating the life processes in the living organisms. As an essential trace element copper acts as a cofactor in multiple enzymes, including superoxide dismutase (SOD), ceruloplasmin, Cu monooxygenases, cytochrome c oxidase, etc. At the same time, copper is toxic to microorganisms and may lead to their death even within minutes of their exposure to copper.[14] The toxic effect may involve inhibition of growth,[15,16] substitution of essential ions and blocking of functional groups on proteins,[17] inactivation of enzymes,[18,19] disturbances of the metabolism,[20] alterations of membrane integrity and production of ROS.[16,21,22]

Chromium is a relatively abundant element in the Earth's crust. It represents an essential micronutrient for living organisms as a participant in the maintenance of normal carbohydrate metabolism in mammals and yeasts.[23] Moreover, it has also been suggested that Cr(III) is involved in the tertiary structure of proteins and in the conformation of cellular RNA and DNA.[24] At the same time, chromium pollution caused serious problems in many regions of the world. It is a transition metal, water soluble, enters living cells easily and is toxic and carcinogenic. Trivalent (Cr[III]) and hexavalent (Cr[VI]) compounds are thought to be the most biologically significant.[25] Inside living cells, the redox cycling of chromium species in different oxidation states generates ROS via the Fenton and Haber–Weiss reactions.[26]

Cadmium is a prevalent non-essential, redox-inactive, highly toxic metal. It is an important heavy metal pollutant. Cadmium shows high affinity towards functional groups of biomolecules, i.e., amino, carboxyl, phosphate and thiol groups,[27] and interferes with numerous biochemical and physiological processes like photosynthesis, respiration, plant–water relationships, nitrogen and protein metabolism, and nutrient uptake.[28] There are some evidences that cadmium-induced oxidative stress in *Saccharomyces cerevisiae*, since strains deficient in antioxidant defence enzymes have a high sensitivity to cadmium and cells grown in the absence of oxygen are more tolerant to cadmium.[29] The bacterial response to cadmium includes induction of expression of genes in many regulons, including genes involved in metal transport, DNA repair, the heat shock response and the oxidative stress response (see [29]).

Despite the great interest in the microbial metal bioaccumulation, the mechanism of oxidative stress induction by heavy metals with different redox status, particularly redox-inactive metals, is not fully understood.

Therefore, the aim of present paper was to evaluate and compare the effect of redox-active (copper and chromium ions) and redox-inactive (Cd) metals on induction of oxidative stress events in *T. cutaneum* R57 cells. To do this, we determined the growth, ROS production and oxidative damaged protein content in yeast cells exposed to different concentrations of metal ions for 6 h. In addition, the role of antioxidant enzymes SOD and catalase (CAT) in the cell response was investigated.

## Materials and methods

### Yeast strain and culture conditions

The *T. cutaneum* R57 strain was obtained from National Bank of Industrial Microbial and Cell Cultures, Bulgaria. The basidiomycete yeast strain of *T. cutaneum* R57 has been registered under N2414.[30]

The cultivation was performed as follows: 80 mL of seed medium was inoculated with 5 mL preculture at a concentration of 2 × 10^8^ CFU/mL in 500 mL Erlenmeyer flasks, on a shaker (220 rpm) at 28 °C for 24 h. Then different concentrations of CdSO_4_ (1, 5 and 10 mmol/L), K_2_Cr_2_O_7_ (1, 5 and 10 mmol/L) and CuSO_4_.5H_2_O (0.5, 1 and 3 mmol/L) ions were added to the culture medium and cultivation continued for the next 6 h. These concentrations were chosen because they allow us to obtain enough biomass for bioaccumulation experiments.

### Cell-free extract preparation

The cell-free extract was prepared as described earlier.[31] All steps were performed at 0–4 °C.

### Enzyme activity determination

SOD activity was measured in cell-free extract by the nitro-blue tetrazolium (NBT) reduction method.[32] One unit of SOD activity was defined as the amount of SOD required for inhibition of the reduction of NBT by 50% (A560) and was expressed as units per mg protein (U/mg protein). Catalase was assayed by the method of Beers and Sizer,[33] in which the decomposition of H_2_O_2_ was analysed spectrophotometrically at 240 nm. One unit of catalase activity was defined as the amount of enzyme that decomposes 1 mmol H_2_O_2_ min^−1^ at an initial H_2_O_2_ concentration of 30 mmol/L at pH 7.0 and 25 °C. The specific activity is given as U/mg protein.

### Determination of ROS

For measurement of ^•^O_2_^−^ production rate, the method of SOD inhabitable reduction of cytochrome c was used.[34] A molar extinction coefficient of 2.11 × 10^4^ was used to calculate the concentration of reduced cytochrome c.

For measurement of hydrogen peroxide production, the method of Pick and Mizel [35] was used. For calculations, a standard curve with H_2_O_2_ concentrations (from 5 to 50 mmol/L) was used.

### Measurement of protein carbonyl content

Protein oxidative damage was measured spectrophotometrically as protein carbonyl content using the 2,4-dinitrophenylhydrazine (DNPH) binding assay,[36] slightly modified by Adachi and Ishii.[37] Following metal treatment, the cell-free extracts were incubated with DNPH for 1 h at 37 °C; proteins were precipitated in 10% cold trichloroacetic acid and washed with ethanol:ethylacetate (1:1), to remove excess DNPH and finally dissolved in 6 mol/L guanidine chloride, pH 2. The optimal density was measured at 380 nm, and the carbonyl content was calculated using a molar extinction coefficient of 21 (mmol/L)^−1^ cm^−1^, resulting in final measurement of nanomoles of DNPH incorporated (protein carbonyls) per mg of protein.

### Determination of reserve carbohydrates

In order to determine glycogen and trehalose content, a procedure previously described by Becker [38] and Vandercammen et al. [39] and then modified by Parrou and Francois [40] was used. Soluble reducing sugars were determined by the Somogyi–Nelson method.[41]

### Other analytical methods

Protein was estimated by the Lowry procedure [42] using crystalline bovine albumin as a standard.

Microbial growth was monitored by measuring the dry weight using Electronic Moisture Balance (KERN, Germany).

## Results and discussion

### Effect of metal ions on growth

The effects of heavy metals on yeast cell growth mainly depend on the mechanisms of metabolic or passive uptake of toxic ions into the cells. The highest concentrations of the metals used are chosen in our preliminary investigations as limiting for the organism survival (data not shown).

**Figure 1.**
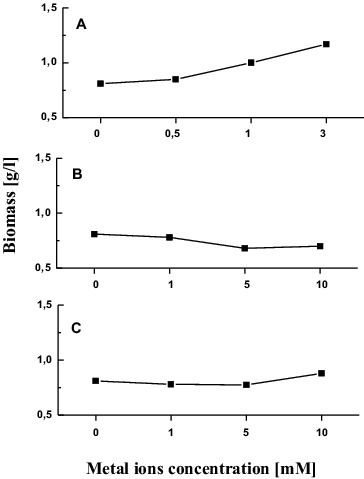
Growth of *T. cutaneum* R57 cells in the presence of different concentrations of metal ions (A) Cu, (B) Cr, and (C) Cd.

Growth of *T. cutaneum* R57 was studied in relation to 0–3 mmol/L CuSO_4_.5H_2_O exposure under submerged conditions (Figure 1(A)). Presence of copper ions in cultural medium induced yeast growth. Mycelia weight increased with rising of metal concentrations. The highest biomass production was detected at a concentration of 3 mmol/L (167% compared with the control).

As shown in Figure 1(B), chromium concentrations of 5 and 10 mmol/L led to 16% less biomass production than the control. Slight reduction of the dry weight at 1 and 5 mmol/L was observed as a result of yeast cell treatment with cadmium ions. Exposure to the next concentration used (10 mmol/L Cd) caused only a minor increase (8%) in biomass yield compared to the control (Figure 1(C)).

### Effect of metal stress on ROS generation

In the present experiments, all heavy metal concentrations applied to yeast culture induced oxidative stress events. It should be noted that the presence of copper, chromium or cadmium ions drastically changed ROS level in *T*. *cutaneum* R57 cells. Table 1 shows the effect of copper, chromium and cadmium ions on **^·^**O_2_^−^ and H_2_O_2_ production in *T. cutaneum* R57 cells after 6 h of exposure to the metal ions. Elevation in **^·^**O_2_^−^ content was found for all metal concentrations tested.

**Table 1.** Increase in ROS generation in the intact cells of *T. cutaneum* R57 treated by enhanced concentrations of Cu, Cr and Cd ions.

	Cu		Cr		Cd	
Variants	**^·^**O_2_^−^ (nmol /mg d.w./h)	H_2_O_2_ (μmol /L.mg d.w./h)	**^·^**O_2_^−^ (nmol/mg d.w./h)	H_2_O_2_ (μmol/L.mg d.w./h)	**^·^**O_2_^−^ (nmol/mg d.w./h)	H_2_O_2_ (μmol/L.mg d.w./h)
Control	0.63	13.1	0.60	15.20	0.69	14.00
0.5 mmol/L	0.65	13.1	Nd	Nd	Nd	Nd
1 mmol/L	0.89	22.4	0.90	33.9	0.86	20.80
3 mmol/L	1.15	21.6	Nd	Nd	Nd	Nd
5 mmol/L	Nd	Nd	1.58	37.5	1.20	22.00
10 mmol/L	Nd	Nd	1.80	28.6	2.41	25.6

As is apparent from Table 1, the treatment by copper ion concentrations in the range from 0.5 to 3 mmol/L resulted in gradual increase in **^·^**O_2_^−^ level above 0.5 mmol/L. The maximum response was achieved with 3 mmol/L (182% compared with the control).

Short-term exposure to 1 and 5 mmol/L chromium ions increased the **^·^**O_2_^−^ level by about 1.5 and 2.7-fold, respectively, as compared to the control. The cells treated with 10 mmol/L showed extremely high **^·^**O_2_^−^ levels (threefold higher than controls).

Cadmium ions had a similar effect on **^·^**O_2_^−^ production. The level of **^·^**O_2_^−^ increased depending on the metal concentrations. Maximum superoxide production was observed by the treatment with 10 mmol/L cadmium ions (349% in comparison with the control).

Metal treatment also caused an increase in H_2_O_2_ levels in concentration-dependent manner. A significant induction in H_2_O_2_ production was observed after 6 h of incubation with copper ions (1.6- and 1.7-fold higher in comparison with the control at concentrations of 1 and 3 mmol/L, respectively). Exposure to 1, 5 and 10 mmol/L chromium ions led to a significant increase in H_2_O_2_ levels compared to the control (2.2-, 2.5- and 1.9-fold, respectively). The same trend was shown for the Cd ions – 1.4-, 1.6- and 1.8-fold increase after exposure to 1, 5 and 10 mmol/L, respectively.

The current experiments indicated that metal treatment significantly increased production of ^·^O_2_^−^ and H_2_O_2_. This increase did not depend on the type of metals (redox-active or redox-inactive) but depended on their concentrations. Even a small increase of metal concentration in the cell led to ROS generation. Redox-active metals, such as copper and chromium, are prone to participate in the formation of ROS via a Fenton-like reaction.[25] Similar direct analyses of ROS content in microbial cells have not often been reported. Transition metal copper is one of the most potent elements catalysing Fenton's reaction. Copper treatment induced ROS generation in yeasts *S. cerevisiae* [43] and fungi *Podospora anserina*,[44] *Humicola lutea* [16] and aquatic hyphomycetes *Varicosporium elodeae* and *Heliscus submersus*.[45] Similar results have been reported for several plants [46] and human cells.[47,48] Chromium ion exposure also induced ROS generation in yeasts, fungi [49] and plants.[50] The redox-inactive metal cadmium affected ROS production in the model strain *T*. *cutaneum* R57 in the same way as copper and chromium. Cadmium, as the least representative member of the transition element group, does not induce production of ROS though a Fenton-like redox cycling mechanism, as is the case with copper and chromium.[51] However, this metal inhibits mainly complexes II and III of the electron transport chain and this inhibition induces ROS generation in the mitochondria. We found that cadmium induces ^·^O_2_^−^ and H_2_O_2_ about 3.5- and 1.9-fold, respectively, as compared to the control. This supports previous studies that suggested possible role of elevated ROS production in mediating Cd toxicity to bacteria,[52] yeast [53] and plants.[54]

### Metal exposure caused protein oxidation

**Figure 2.**
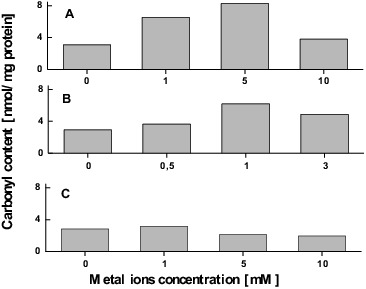
Protein oxidation induced in *T. cutaneum* R57 cells by Cu (A), Cr (B) and Cd (C) ions.

As a consequence of excessive ROS production, oxidative damages of proteins occurred.[55] Protein carbonylation, one of the most harmful irreversible oxidative protein modifications, is used as a biomarker of metal-induced oxidative stress. Exposure to elevated copper concentrations did not significantly affect carbonyl content with exception of 1 mmol/L when the carbonylated protein showed 42% higher level than in the control (Figure 2(A)).

In contrast, treatment with enhanced chromium ion concentrations up to 5 mmol/L caused significant increase in the protein carbonyl content (about 2.3-fold compared with the control). It should be noted that the subsequent concentration (10 mmol/L) resulted in a sharp decrease in the amount of oxidative damaged protein (Figure 2(B)).

When the *T. cutaneum* R57 cells were treated with cadmium ions, the trends of protein carbonyl content showed a similar profile as after treatment with copper ions, and the highest level of damaged protein was found at 1 mmol/L concentration (Figure 2(C)).

The results mentioned above indicate that exposing *T*. *cutaneum* R57 to elevated concentrations of copper, chromium and cadmium ions inflicts oxidative damage on the intracellular proteins. It is worth noting that the redox-active metal ions (copper and chromium ions) demonstrated more drastic changes in oxidative damaged protein content. The majority of proteins that were oxidized over the course of the investigation demonstrated an increase in their relative abundance after exposure to 1 mmol/L CuSO_4_.5H_2_O, 5 mmol/L K_2_Cr_2_O_7_ and 1 mmol/L CdSO_4_. The subsequent decline in abundance after treatment with higher concentrations could be explained by enhancing both degradation of proteins by proteases and aggregation of heavily oxidized proteins (see [16]). Evidence for a positive correlation between increased levels of ROS and damaged proteins has been published for various microbial cells.[16]

### Effect of stress on glycogen and trehalose content

**Figure 3.**
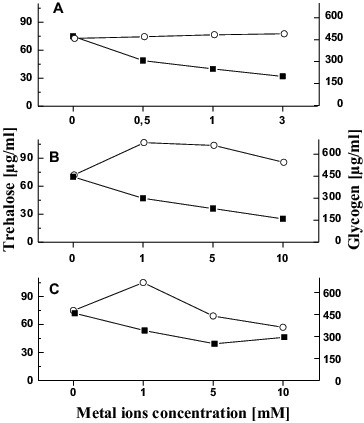
Changes in the glycogen (-o-) and trehalose (-▪-) level in *T. cutaneum* R57 cells treated with Cu (A), Cr (B) and Cd (C) ions.

The metal exposure of *T. cutaneum* R57 was accompanied by quantified changes in the reserve carbohydrates such as trehalose and glycogen. Level of reserve carbohydrates was influenced by all the metal ions added (Figure 3). We observed reduction of trehalose amount after 6 h of metal treatment. This reduction was the most significant in the variant treated with 10 mmol/L Cr (65% lower than the control) (Figure 3(B)).

The other carbohydrate glycogen showed a different behaviour. The glycogen amount in *T. cutaneum* R57 was not influenced after 6 h of copper treatment (Figure 3(A)).

We observed an increase in the level of glycogen in the other variants tested. Experiments with chromium ions showed a trend of enhancing this carbohydrate (above 48%) in variants with 1 and 5 mmol/L and then a slight decrease similar to the control level (Figure 3(B)). A similar trend is observed in variants with cadmium ions. After a 40% increase in glycogen level, a sharp decrease was observed at 10mmol/L Cd (Figure 3(C)).

As was mentioned above, the metal exposure of *T. cutaneum* R57 results in changes of the reserve carbohydrates such as trehalose and glycogen. Both compounds have different physiological effects and mode of action: trehalose might be a more general stress protectant and assists chaperones in controlling protein denaturation and renaturation, and glycogen is a storage carbohydrate.[56,57] Our results indicated that chromium and cadmium ion treatment led to a significant increase in glycogen content. These findings agree with earlier studies about the microbial response against heavy metal stress including chromium and cadmium ions.[21] In contrast, no changes were found in the amounts of glycogen in the cells treated with copper ions. Similar data has been reported by stress response in yeasts.[58] Furthermore, *Arapaima gigas* (a carnivorous fish) and the fry of common carp demonstrated a decrease in glycogen level under conditions of copper stress.[59,60] The possible explanation is a progressive glycogenolysis (breakdown of glycogen (*n*) to glucose-1-phosphate and glycogen (*n* − 1)) in the cells.[61]

In addition, the trehalose amount gradually decreased with increase in the metal ion concentration. These results could be due to the active metabolism of trehalose, its simultaneous synthesis and degradation during the period of stress.[62] Probably, after 6 h of exposure to metal stress neutral trehalase (enzyme responsible for trehalose degradation) exhibits higher activity than trehalose 6-phosphate phosphatase (enzyme responsible for its synthesis).

### Metal effect on SOD and CAT activity

**Figure 4.**
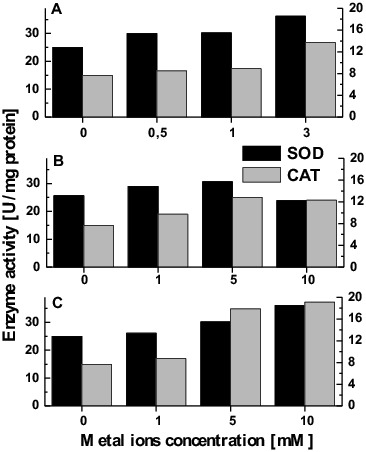
Antioxidant response of *T. cutaneum* R57 against metal-induced stress (A) Cu, (B) Cr, and (C) Cd.

Fungi, like all aerobic organisms, have a set of defence mechanisms to deal with oxidative stress.[63] The mechanism of metal-induced formation of ROS is strongly modulated by the action of cellular antioxidants. The presence of metal ions stimulated enzyme antioxidant defence in *T. cutaneum* R57 cells. The results shown in Figure 4(A) demonstrate a gradual elevation in SOD activity after exposure to enhanced concentrations of copper ions. The maximum activity was observed at the concentration of 3 mmol/L when about 1.5-fold higher specific enzyme activity was achieved. While the treatment of yeast cells with 1 or 5 mmol/L chromium caused an insignificant increase in the level of SOD (19% compared to the control), the next concentration (10 mmol/L) resulted in a reduction of 22% compared with the highest enzyme activity (Figure 4(B)). The activity of SOD raised dose-dependent manner in a variant of cadmium ions treatment. The highest enzyme activity was observed at the concentration of 10 mmol/L (Figure 4(C)).

As shown in Figure 4, the effect of metal treatment was more pronounced for CAT than for SOD. All of the metal ions tested increased CAT activity in a concentration-dependent manner. The maximum enzyme activity was 1.8-, 2.5- and 1.6-fold higher for copper, chromium and cadmium ions, respectively, than the activity in the corresponding control variant.

Enzymes, such as SOD and CAT, have been reported to be activated as a result of elevated ROS levels in several organisms exposed to heavy metal stress.[16,64] These enzymes are crucial for cellular detoxification, controlling the levels of superoxide anion radical and hydrogen peroxide.[63] Higher activities of SOD and CAT are associated with the induced resistance of the mycetes to different stress factors.[65,66] Our result indicated that all the metals used induced activation of the enzymes involved in antioxidant defence. Treatment of yeast cells with copper, chromium and cadmium ions showed a clear tendency of concentration-dependent stimulation of SOD and CAT activity.

## Conclusion

In summary, our results provide additional confirmation for metal-mediated oxidative stress in filamentous yeasts. Both redox-active and non-redox-active metals caused oxidative stress events which included enhanced levels of oxidatively damaged proteins, changes of glycogen and trehalose levels, and activation of the antioxidant enzymes SOD and CAT. Despite the significant induction of antioxidant enzyme activity, copper exposure still has deleterious effects, probably mediated by the overloading of antioxidant defences.
